# IEEN workshop report: Professionalism in interdisciplinary and empirical bioethics

**DOI:** 10.1177/1477750914558550

**Published:** 2014-12

**Authors:** John Owens, Jonathan Ives, Alan Cribb

**Affiliations:** 1 Centre for Public Policy Research, King’s College London, London, UK; 2 MESH, The University of Birmingham, UK

**Keywords:** Professional ethics, education, philosophical aspects, codes of ethics

## Abstract

The Interdisciplinary and Empirical Ethics Network was established in 2012 with funding from the Wellcome Trust in order to facilitate critical and constructive discussion around the nature of the disciplinary diversity within bioethics and to consider the ongoing development of bioethics as an evolving field of interdisciplinary study. In April 2013, the Interdisciplinary and Empirical Ethics Network organized a workshop at the Centre for Public Policy Research, King’s College London, which discussed the nature and possibility of professionalism within interdisciplinary and empirical bioethics. This paper provides a report of that workshop.

Bioethics is a diverse, interdisciplinary field that accommodates a broad range of perspectives and traditions. In 2012, the Interdisciplinary and Empirical Ethics Network (IEEN) was established with funding from the Wellcome Trust to facilitate critical and constructive discussion around the nature of this disciplinary diversity and the ongoing development of bioethics as an evolving field of interdisciplinary study. In April 2013, the IEEN's third workshop was held at the Centre for Public Policy Research, King’s College London, to consider issues associated with professionalism in interdisciplinary and empirical bioethics. The workshop focused on bioethicists’ occupation of professional roles, either at institutions or in association with bodies authorised by professional groups, and sought to explore how these roles are socially constructed, the ethical dimensions attached to the performance of these professional roles, and how performance of these roles may be judged and evaluated. Speakers and attendees were asked to consider the following core questions: (1) What does it mean to be a good bioethicist, and what expertise is required? (2) According to what standards should a professional bioethicist be judged? (3) What authority and influence do bioethicists have, and how might any potential authority and influence be recognised, regulated and evaluated? With these questions in mind, the morning session considered perspectives on professionalism in and from bioethics, while the afternoon session was loosely directed towards the question of whether professional standards in bioethics could be institutionally underpinned.

Søren Holm (University of Manchester) gave the first presentation of the day with a talk that began by asking whether there could in fact be a ‘profession’ of bioethics. He noted that, whilst standards and expertise are important, when we think about professional standards we often also think about norms of behaviour. He asked the question ‘why might we, as bioethicists, need a profession’, and suggested that one reason may be that it offers some kind of security and validation of our work and our views: being a ‘professional’ is better than being an ‘amateur’. Considering the various activities in which bioethicists tend to be engaged, including their roles as researchers, teachers, experts on committees and/or public intellectuals and campaigners, Søren suggested that the extent to which professionalism may be applied to each of these roles or activities, as well as the nature of professionalism in each case, depends on the precise nature of the role or activity in question. Given the diversity of activities undertaken by bioethicists, Søren was not confident that the notion of professionalism could successfully and/or straightforwardly be applied to bioethics in general. He pointed out that each of these varied roles demand a diverse range of skills and knowledge, including conceptual, regulatory and legal knowledge, as well as analytic, problem solving, interpersonal and communicative skills. Any notion of a professional expertise in bioethics would need to be broad enough to encompass this spread of skills and knowledge.

In an attempt to move beyond the complexities associated with such diversity, Søren turned his attention specifically to professionalism in the case of the empirical bioethics researcher, suggesting that they ought to be: (1) capable of understanding and executing the appropriate empirical and analytic methods of empirical bioethics in order to produce rigorous, high-quality work; (2) aware of the relevant standards and requirements for bioethics research as well as potentially related subject areas and/or disciplines; (3) aware of the context in which their research will be published and/or applied and be able to contextulize their findings in relation to further important ethical questions. Søren went on to express scepticism about the prospect of there ever being a profession of ‘empirical bioethicist’. Rather, empirical bioethics is something that a bioethicist might do. He concluded that whilst it may be possible to use broad categories to judge professional practice in bioethics on a specific case by case basis, such evaluations seem vague, making it unlikely that a general standard for understanding and evaluating the role of the professional bioethicists could be successfully drawn up.

Søren’s presentation was followed by Richard Ashcroft (Queen Mary's University of London), who began by declaring that he is not a bioethicist, despite holding a professional role as a bioethicist and being widely recognised as such. He pointed out that professional roles are socially attributed, making the occupation of such a role at least partially an issue of meeting the expectations of others. Given the inherent interdisciplinarity within the field of bioethics, Richard highlighted how occupying a professional bioethics role could be particularly challenging, especially if this requires one to conform to a variety of paradigms, perspectives, traditions and standards. He thus described professionalism within bioethics as a kind of ‘bricolage’, in the sense that one may need to draw upon a diverse array of resources and techniques in order to perform as expected. However, despite the need to work across disciplinary boundaries, Richard suggested that working as a bioethicist within the academy requires a clear set of professional qualities, associated with being a professional academic more generally rather than a bioethicist in particular: producing work on time; reviewing work fairly; not speaking of the unknown; teaching to a certain standard; conducting oneself as a scholar. Beyond the professional role that accompanies an academic position, Richard suggested that bioethicists ought not to seek to become a profession in the formal sense, and that formal professional status is neither required nor desirable.

The discussion that followed centred on what it might mean to perform the role of a bioethicist ‘well’, as a proxy for ‘professionally’. John Owens began by asking whether interdisciplinarity would be a prerequisite for professionalism in bioethics, and this was answered in the negative by Søren, who argued that this would be too limiting. Richard supported this, suggesting that being interdisciplinary cannot be necessary to participate in professional bioethics. Rather, people need to be good at one particular thing, and be able to work with people from other disciplines where necessary. Discussion then turned to the question of how we can tell if bioethics is done well, and Jon Ives, playing Devil’s advocate, suggested that, at least to some degree, the peer-review process acts as a mechanism for ensuring that professional standards have been met in the context of bioethics research, and that this might be considered as a form of informal self-regulation. In response, a number of problems with the peer-review process as a model for self-regulation were considered by the group: reviewers’ attitudes can vary between being overly critical and overly sympathetic; there are a number of journal publications within the field of bioethics spanning a variety of genres, styles, disciplinary orientations and readerships, making it difficult to find standard criteria for good research in bioethics; there is not yet an established canon of literature in empirical and interdisciplinary bioethics against which standards could be judged.

The discussion then moved on to consider the power of bioethics professionals, with the suggestion from Suzanne Shale that it may be possible to identify an outline of the bioethics profession through the specific interests that they assert. Søren Holm suggested that the development of a profession in bioethics might be seen to emerge through the development of sociological and philosophical critiques of powerful medical and legal discourses and practices; and whilst this picture may account for the emergence of bioethics as a community, it does not support calls for recognising bioethics as a profession. In response to aired concerns that bioethics lacks a common aim, methodology and forms of evaluation and regulation, and may therefore be too broad and nebulous to be called a profession, Alan Cribb suggested that bioethics may plausibly be thought of as a label which describes a collective group of diverse sub-professions. On this reading, bioethics would not be considered a profession in the formal sense, but the question about the power attached to the role of bioethicist remains important. Picking up on the idea of sub-professions, Jon Ives then noted that a perennial problem for professionalism, in the context of bioethics, may be that there is a sense in which there is no need to have specialists in bioethics, given that the roles bioethicists play (that of commenting on ethical issues and critiquing practice) can arguably be taken on by anyone with an interest in those issues. The need for a profession, and for a recognition of disciplinary expertise for the bioethicist, is one unique to the academy, and not a concern for wider society. Richard Ashcroft agreed that all medical professionals are supposed to routinely engage with bioethics, and suggested that their willing and respectful attitudes towards bioethics may be more important than specialist bioethical knowledge.

The afternoon session began with a presentation by Suzanne Shale, an ethics consultant and research associate at The University of Oxford’s ETHOX Centre. Suzanne presented her ethics consultancy work as an example of how professional bioethics can be performed beyond academic and clinical contexts. Suzanne began by exploring how moral life is different from moral research. In real life we negotiate moral demands through narratives of value, relationships and identity, where normative expectations regulate our behaviour and we have strong reactive attitudes when these expectations are breached. Given the dynamic nature of our moral experience, Suzanne questioned what it is that the professional bioethicist claims to know and to do, and the domain in which this tends to occur. She suggested that the domain of professional bioethics tends to be one of expert reasoning about the moral literature and social fields of law, education, policy and research, and that their core function tends to involve providing criticism, guidance and support. Suzanne’s approach to bioethics is less typical, and does not conform to this professional orthodoxy. She describes herself as working in the domain of moral dialogue, with a focus on the emotional intensity associated with moral norm expectations and a function of providing space for conversations about the moral experience for those who require this sort of support. She explained that her work often focuses on processes of moral repair for people who have been harmed by medical institutions by providing a means of exploring experiences of harm through moral dialogue. Drawing on Walker’s^1^ work on moral repair and Berlinger’s^2^ studies on the ethics of forgiveness, Suzanne uses literature from bioethics to create spaces for moral conversations about grief, healing and repair to take place which have a positive impact on those with which she works. Suzanne’s account testifies to the diverse and varied nature of professional roles within bioethics.

Suzanne was followed by Hugh Whittall, director of the Nuffield Council on Bioethics (NCB). In his presentation, Hugh identified interdisciplinary ethics as important to the NCB’s work, which he described as typically identifying ethical issues to which value based, empirical and analytical reasoning processes could be applied to reach conclusions for policy application. He suggested that professionalism in the institutional context of the NCB might comprise having sufficient knowledge and expertise, as well as having the ability to engage in appropriate behaviour to meet expectations. Hugh identified a variety of potential professional roles for bioethicists: (1) decision-making roles which might take place in a group or an institutional context, but may also be undertaken as an individual (whether as a patient, professional, academic, etc.) embedded within a social context; (2) expert roles which might involve providing support, critique, advice or guidance; (3) practical policy roles which might involve implementing a decision that has been made, and could mean taking into account guidance from experts. Hugh’s central point was that establishing the nature, purpose and conditions of a role is key for understanding what professionalism may mean in any given context. Hugh suggested that the NCB’s institutional role is also context dependent, but normally involves identifying and interpreting questions, locating a decision-maker and producing reports which would support decision-making. He identified transparent justification as key to good performance, and explained that this meant making the values and empirical data on which the Council’s reports are based open and available for public scrutiny. In this way, Hugh suggested that the NCB could potentially be seen as a bioethics institution fulfilling a professional role, but this doesn't involve decision-making, underwriting what is correct or underpinning standards within bioethics. Whilst an institution might one day be created to codify standards of practise within bioethics, and thus lend bioethicists a professional status, Hugh concluded by suggesting that the professional status of bioethics is less important than ensuring that bioethics is practised to a high standard. As such, institutions like the NCB can be a useful mechanism for supporting high-quality bioethics activity through forms of education (for example, by presenting syllabus contents for bioethics courses) or practice (for example, by engaging in and/or promoting certain forms of behaviour). However, Hugh acknowledged that these measures may not be sufficient for ensuring high-quality outcomes in bioethics; indeed we may be worried that they might promote certain forms, discourses and practices of bioethics at the expense of other valuable elements. For this reason, the potential limitations of institutions contributing to bioethics ought to be recognised alongside their potential strengths and resources.

In the discussion that followed, Suzanne was asked to clarify the difference between her work in moral repair and therapy. She replied that whilst there are similarities, particularly insofar as there is a one-to-one focus, the difference is in the subject matter. Her work very much centres on having a moral conversation. Hugh Whitall then noted that we all make moral decisions every day and, by and large, we do this well, often without really thinking and without making our reasoning explicit. When the problems get complex we tend to struggle, and this is when some expertise or ‘professional’ input might be useful. The implication here is that the ‘professional bioethicist’, whether doing the work that Suzanne described, or the work that the NCB undertakes, can bring something unique and valuable to the table, in terms of skills and experience. Whether this ‘professional’ is a single individual, or a group of people, will depend on the context of the problem. Picking up on an earlier point from Søren’s talk, John Owens then suggested that given the divergent nature of these bioethics ‘tasks’, it might be insufficient to use any kind of institutional guidance to determine what it is to be a bioethics ‘professional’, as those kinds of rules could never capture the complexity of the roles. Rather, professionalism might be carved out in contested spaces, and defined in practice rather than outside of it. Angus Dawson then took the discussion back to the afternoon talks, and asked (1) what work is the word ‘moral’ doing in Suzanne’s concept of ‘moral harm’? and, (2) how does the impact agenda get into the NCB’s work and it’s professional identity? Hugh replied, stating that the NCB does not want to idealise its role. There are various kinds of impact, ranging from getting people do what the Council suggests to simply contributing to a discussion. The NCB attempts to do all of this, and does not consider itself the final authority on matters bioethical. Coming back the point he made in his talk about a marker of professionalism, he noted that the NCB tries to make all of its reasoning very explicit, and that makes impact, of different kinds, possible (rather than guaranteed). Suzanne then replied to Angus’ question by saying that she was not entirely sure what made something a ‘moral’ issue specifically, but suggested that wider interpretation is needed than what seems to be used currently in the academy: we tend to think about ethics exclusively in terms of the question ‘what do we do next?’, rather than asking ‘what do we do when we have stuffed things up’? The concept of ‘moral repair’ is tied to the latter, and is not just concerned with relations between individuals, but also with how an individual can reconcile and accept moral harm done to them themselves.

## Summary

The meeting was successful in its aim of bringing together members of the bioethics community to explore questions of professionalism within bioethics. Whilst no firm conclusions were reached, the presentations and subsequent discussion highlighted the importance of: (a) unpacking the variety of activities which bioethicists undertake and the diverse contexts in which this work takes place; (b) analysing roles, expertise and standards in bioethics in relation to this variety of activities and diversity of contexts; (c) debating the benefits that professionalisation within bioethics may bring against the challenges and potentially negative consequences of such efforts.

A report on the fourth IEEN workshop, which was held in Birmingham in late 2013 on the theme of Dissemination, Publication & Impact in Interdisciplinary and Empirical Ethics, is forthcoming. Details of future events will be posted on the website and circulated to network members. To get involved in the network, or for more information, please contact the authors of this paper or visit the network website (http://www.birmingham.ac.uk/research/activity/mds/projects/HaPS/PCCS/MESH/ieen/index.aspx).
